# A prospective real-world analysis of erenumab in refractory chronic migraine

**DOI:** 10.1186/s10194-020-01127-0

**Published:** 2020-06-01

**Authors:** Giorgio Lambru, Bethany Hill, Madeleine Murphy, Ivona Tylova, Anna P. Andreou

**Affiliations:** 1 The Headache Centre, Pain Management and Neuromodulation Centre, Guy’s and St Thomas NHS Foundation Trust, London, UK; 2 Headache Research-Wolfson CARD, Institute of Psychology, Psychiatry and Neuroscience, King’s College London, London, UK

**Keywords:** Erenumab, Chronic migraine, Refractory migraine, CGRP, Monoclonal antibodies

## Abstract

**Background:**

Clinical trials have shown the safety and clinical superiority of erenumab compared to placebo in chronic migraine (CM). The aim of this analysis is to evaluate the effectiveness and tolerability of erenumab in a real-world setting in patients with refractory CM.

**Methods:**

This is a prospective single centre real-world audit conducted in patients with CM with and without medication overuse, refractory to established preventive medications, who received monthly erenumab for 6 months.

**Results:**

Of 164 patients treated, 162 patients (female = 135, mean age 46 ± 14.3 years old) were included in the audit. Patients had failed a mean of 8.4 preventive treatments at baseline and 91% of patients failed Botulinum toxin type A at baseline. The mean reduction in monthly migraine days was 6.0 days at month 3 (*P* = 0.002) and 7.5 days at month 6 (*P* < 0.001) compared to baseline. The mean reduction in monthly headache days was 6.3 days (*P* < 0.001) at month 3 and 6.8 days (*P* < 0.001) at month 6. At month 3, 49%, 35% and 13% and at month 6, 60%, 38% and 22% of patients obtained at least a 30%, 50% and 75% reduction in migraine days, respectively. The percentage of patients with medication overuse was reduced from 54% at baseline to 20% at month 3 and to 25% at month 6. Compared to baseline, the mean reduction of Headache Impact Test-6 score was 7.7 points at month 3 (from 67.6 ± 0.4 to 59.9 ± 0.9) (*P* < 0.001) and of 7.5 points at month 6 (60.1 ± 1.3) (*P* = 0.01). The percentage of patients with severe headache-related disability (HIT-6: 60–78) was reduced from 96% at baseline to 68% after three monthly treatments and to 59% after six treatments. At least one side effect was reported by 48% of patients at month 1, 22% at month 3 and 15% at month 6. Constipation (20%) and cold/flu-like symptoms (15%) were the most frequent adverse events reported.

**Conclusion:**

Erenumab may be an effective and well tolerated therapy for medically refractory CM patients with and without medication overuse.

## Introduction

Chronic migraine affects 1.4–2.2% of the general population with an annual incidence among episodic migraine people of 2.5% [1, 2]. The combination of daily or nearly daily head pain, other comorbidities, namely psychiatric, sleep and pain related, along with the frequent association with medication overuse headache (MOH), contributes to the high degree of socioeconomic burden typical of this condition [3]. The cornerstone strategy to reduce symptoms in CM includes preventive treatments, however a significant minority of CM patients fails to respond or tolerate numerous preventive treatments [4]. Refractory CM is still a debated definition, but it essentially refers to the group of difficult-to-treat CM patients who fail to respond/tolerate at least two or three classes/medications amongst the ones considered effective in migraine prevention [5, 6]. A recent consensus of the European Headache Federation (EHF) has proposed a distinction of the difficult-to-treat migraine patients into resistant, for those who fail to respond or tolerate three drug classes with established evidence in migraine; and refractory, for those patients who fail all drug classes with established evidence in migraine [7]. This group of patients suffers with severe disruption of their quality of life and the refractoriness of their symptoms contributes to high degree of healthcare resources utilization [8, 9]. A vast unmet need for novel effective and well tolerated preventive treatments remains for these patients.

Monoclonal antibodies (MABs) targeting the calcitonin gene related peptide (CGRP) or its receptor, have been approved by the Food and Drug Administration (FDA) for the prevention of symptoms in episodic and chronic migraine in adults [10–12]. However, to date, there is sparse real-world data on the efficacy of the CGRP MABs, especially in the medically refractory group of chronic migraine patients.

In September 2018 Erenumab (Aimovig™), a CGRP receptor monoclonal antibody, was made available free-of-charge in the United Kingdom (UK) for the prevention of CM in patients who failed at least three preventive treatments, as part of an agreement between Novartis and the National Health System (NHS) Trusts across the UK. The agreement would allow the treatment of CM patients with Erenumab until the National Institute for Health and Care Excellence (NICE) published the outcome of their application appraisal. In September 2019, NICE UK decided not to recommend the use of erenumab in the NHS and since then no more new patients were allowed to be treated according to this scheme. Patients who had already started the treatment could continue free-of-charge for further 3 years under the agreement. Here we report our experience using erenumab for refractory CM patients under the above mentioned scheme.

## Methods

This is a registered prospective clinical audit evaluating the effectiveness, safety and tolerability of erenumab in adults with refractory CM. It was part of a service evaluation conducted at the Headache Service at Guy’s and St Thomas’ NHS Foundation Trust, London, UK. New patients were included in the audit between October 2018 and September 2019.

### Participants

Adult patients meeting the International Headache Society (IHS) criteria for CM who failed at least three preventive treatments were included in the audit [13]. These treatments belonged to the following classes: beta-blockers (propranolol, atenolol), tricyclics (amitriptyline and nortriptyline), anticonvulsants (topiramate, gabapentin, pregabalin and sodium valproate), angiotensin II receptor blocker (candesartan), botulinum toxin type A (BoNT/A), bilateral greater occipital nerve blocks (GONBs) calcium channels blockers (flunarizine), serotonin antagonists (pizotifen), serotonin and norepinephrine reuptake inhibitors (SNRI) namely venlafaxine and duloxetine, other antidepressants (mirtazapine) and non- invasive neuromodulation therapies (single pulse transcranial magnetic stimulation).

Treatment failure was defined as treatment discontinuation due to unacceptable side effects and/or absence of reduction in headache frequency, duration or severity after administration of a preventive medication for at least 12 weeks. Contraindicated treatments were not considered as treatment failures. For patients who underwent a trial with botulinum toxin type A (BoNT/A), failure to obtain at least 30% reduction in headache days after two sets of injections was considered treatment failure as per NICE UK guidance [14]. Patients with MOH were not excluded from the audit, since they constitute a significant proportion of difficult-to-treat CM patients in real world settings. When medication overuse was present, withdrawal attempts using outpatients pharmacological and non-pharmacological strategies were tried. Patients were allowed to continue oral preventive medications during treatment with Erenumab.

Audit under current national guidelines does not require research ethics committee review (http:// www.hra-decisiontools.org.uk/research/).

### Audit design

**Fig. 1:**
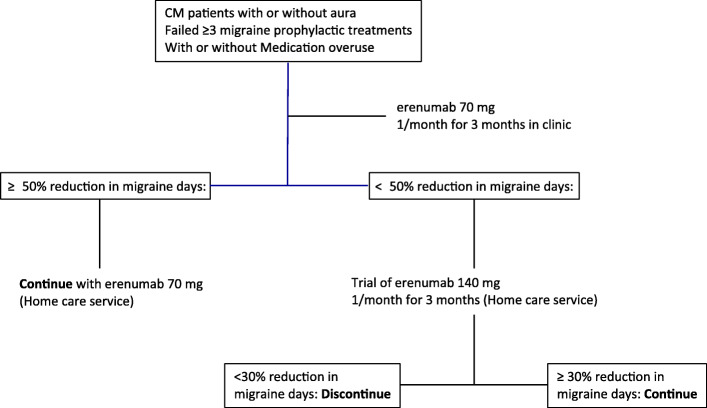
Audit design: Chronic migraine patients who failed at least 3 preventive treatments, with or without medication overused, were offered monthly subcutaneous injections of erenumab at 70 mg for 3 months. At the three-month time point, patients who achieved at least a 50% reduction in migraine days, were offered the option to continue their treatment with monthly injections of erenumab at 70 mg. Patients who achieved less than 50% reduction in their migraine days, were offered the option to receive monthly injections of erenumab at 140 mg for the next three consecutive months. Any patient who achieved less than 30% reduction in their migraine days at the six-month time point discontinued the erenumab treatment, while patients who achieved at least 30% reduction in their migraine days continue the erenumab treatment at 140 mg

Figure 1 outlines the treatment audit profile. Patients were trialled on erenumab for a total of six months before establishing efficacy. All patients received at least three 70 mg Erenumab injections performed one month apart, with the option to increase the dose to 140 mg for further three months if meaningful improvement was not achieved. This treatment paradigm was based upon the lack of definite indications on superiority of 140 mg over 70 mg monthly dose of erenumab, on the most appropriate length of exposure to erenumab in the refractory migraine population and on the high level of refractoriness of our patients. Furthermore, given that our refractory CM population included predominantly BoNT/A non-responders and that under the NICE guidelines two BoNT/A treatments three months apart are recommended before assessing treatment efficacy, it was decided that a 6-month erenumab trial would have fairly assessed erenumab effectiveness in such a complex population.

Patients were demonstrated how to use the pre-filled autoinjector and the subcutaneous injection was administered or supervised by our headache specialist nurses for the first three visits. Blood pressure (BP) and heart rate were measured in clinic at baseline and at every visit for the first three months. Subsequently, delivery of the medication was arranged via a homecare provider. Patients were asked to check their BP every month for the subsequent treatments. Patients were fully informed about the lack of safety data on erenumab during pregnancy. Female patients were asked to promptly inform the clinic in the event of a pregnancy.

### Outcome measures

A migraine-specific diary and the Headache Impact Test-6 (HIT-6) score were used to capture efficacy and disability measures. Patients were required to produce a baseline headache diary and HIT-6 score for at least one month prior to treatment initiation and to continue filling the headache diary on a daily basis along with HIT-6 scores every month for the duration of the trial. Data were entered in an electronic macro database for analysis.

The main efficacy outcomes were changes from baseline in the mean monthly migraine days (MMD) at month 3 and month 6. Secondary efficacy outcomes included: changes from baseline in mean monthly headache days (MHD), change in mean monthly headache-free days, 30%–50%–75% responders (patients who reported respectively a 30%–50%–75% reduction in mean MMD). A “headache day” was defined as a day with headache lasting for ≥4 h and with a severity of ≥4/10 on a verbal rating scale (0, no head pain, 10 worst pain ever experienced). A “migraine day” was defined according to the IHS classification criteria [13]. A “headache-free day” was defined as a day without any head pain. Changes in abortive treatment intake days and change in the proportion of patients with MOH were also evaluated. An “abortive treatment intake day” was considered any day where patients consumed abortive treatments for attempted headache relief. An analysis of outcomes in those patients who increased the dose from 70 mg to 140 mg after the first three months was also performed. The cut-off outcome for free-of-charge treatment continuation was reduction in the mean MMD of at least 30% after six monthly injections. To assess whether any change in efficacy measures was associated with improvement in headache-related disability, change in HIT-6 score were analysed.

Patients were asked about the development of adverse events (AEs) in clinic every month for the first three months, and during telephone follow-ups or clinical appointments for the subsequent months. Adverse events were graded as mild, moderate and severe. The efficacy of erenumab in treated patients was assessed by our specialised headache team every three months for the duration of the trial.

### Statistical analysis

All outcomes pre- and post- erenumab treatment were measured on a continous scale. For all measures considered here, data demonstrated a skewed distribution with a significant deviation from normal distribution (Kolmogorov-Smirnov test; *P* < 0.05). In order to express long term data as a linear graph, skewed data were reflected and transformed to make them more normally distributed [15, 16]. ANOVA for repeated measures was used to compare the change in values over time. Paired t-test was used to compare any time point against baseline data. Independent t-test was used for independent group comparisons. All data are provided as mean (± standard error), unless stated otherwise.

## Results

### Demographic and baseline headache characteristics

A total of 164 patients received at least one injection of erenumab at 70 mg during the audit period. Completed headache diaries and HIT-6 for all months, were obtained by 162 patients [135 female; mean age 46 Standard Deviation (SD) ± 14 years] who were included in the analysis. At the time of analysis for this report, 100 patients received three erenumab injections and 73 patients received six injections. Demographic and clinical characteristics of the patients’ group at baseline are summarised in Table 1. All patients were medically refractory according to the European headache federation (EHF) consensus [5], with the average number of failed preventive treatments being 8.4 ± 3.6. All patients failed to obtain a meaningful response to greater occipital nerve blocks (GONBs). A proportion of 91.4% of patients had also failed to respond to BoNT/A. Forty-one percent of patients reported a daily headache pattern at baseline with no headache-free days. The vast majority of patients (95.7%) were classified in the severe impact category at baseline (HIT-6 score: 60–78).

**Table 1:** Demographic and clinical characteristics at baseline of refractory chronic migraine patients treated with Erenumab

	*Total number of patients*
Sex, M/F	27/135
Age (y), mean ± SD	46 ± 14.3
CM duration (y), mean ± SD	13 ± 11.9
Aura, *N* (%)	53 (33%)
Medication overuse, *N* (%)	87 (54%)
	*Mean ± St. Error*
Migraine days	19.7 ± 0.7
Headache days	23.4 ± 0.6
Headache free days	3.7 ± 0.4
Abortive treatment intake days	11.5 ± 0.7
HIT-6 score	67.6 ± 0.4
Number of preventive treatments failed	8.4 ± 3.6
BoNT/A non-responders	148 (91.4%)

**BoNTA:** onabotulinum toxinA, **CM**, chronic migraine; **F**, female; **HIT-6**, headache impact test-6; **M**, male; **N**, number; **Y**, years

### Efficacy outcomes at month 3 and month 6

**Fig. 2:**
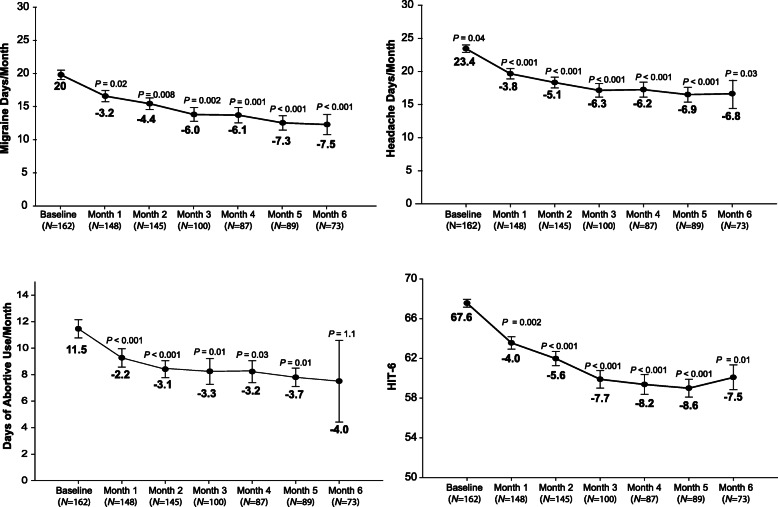
Six-month outcomes on all patients treated with erenumab: Overall, during the entire six month observation period post-erenumab treatment initiation, monthly migraine days (MMD), headache days (MHD), days of abortive use and headache impact test (HIT-6) were significantly reduced compared to baseline (mean ± st. er.)

Overall, during the entire 6-month observation period post-treatment initiation, MMD days and MHD were significantly reduced compared to baseline (MMDs: *F*_4.8, 321.3_ = 3.7, *P* = 0.003; MHDs: *F*_4.9, 326.8_ = 3.5, *P* = 0.005) as well as the number of abortive treatment intake days (*F*_3.6, 240.1_ = 4.5, *P* = 0.002). Additionally, the HIT-6 score was significantly reduced across the entire observational period (*F*_3.8, 116.6_ = 3.8, *P* = 0.007) (Fig. 2).

Compared to baseline, the mean reduction in MMD at month 3 was 6.0 days (from 19.7 ± 0.7 to 13.7 ± 1.0; *P* = 0.002) and at month 6 was 7.5 days (from 19.7 ± 0.7 to 12.2 ± 1.5, *P* < 0.001). The mean reduction in MHD was 6.3 days at month 3 (from 23.4 ± 0.6 to 17.1 ± 1.0; *P* < 0.001) and 6.8 days at month 6 (from 23.4 ± 0.6 to 16.6 ± 1.6, *P* < 0.001) (Fig. 2). Treatment with erenumab also increased the number of headache-free days by 4.2 ± 1.0 days at month 3 (*P* < 0.001) and by 3.0 ± 1.4 days at month 6 (*P* = 0.007). The proportion of patients with a daily headache pattern was reduced from 41% at baseline, to 22% after three and to 21% after six treatments with erenumab. The mean reduction in abortive treatment days was statistically significant at month 3 (3.3 ± 0.7, *P* = 0.01) but not at month 6 (4.0 ± 3.1, *P* = 1.1).

**Fig. 3:**
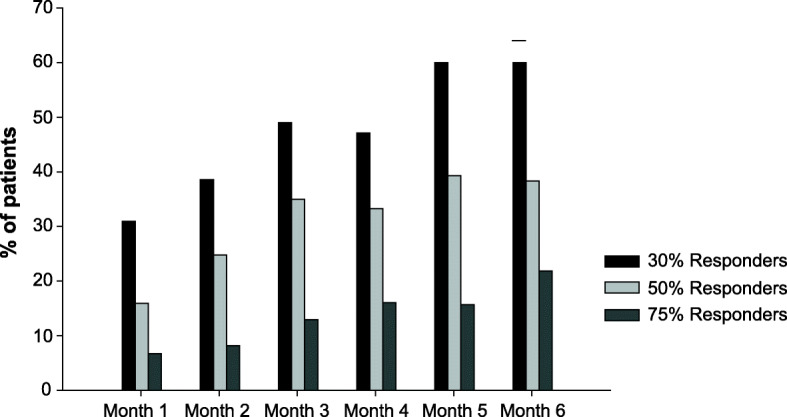
Monthly responders rates: Percentage of patients who achieved at least a 30%, 50% or 75% reduction in monthly migraine days per month, post-erenumab treatment initiation

At month 3, 49%, 35% and 13% out of the 100 patients obtained at least a 30%, 50% and 75% reduction MMD, respectively. At month 6, 60%, 38% and 22% of the 73 patients obtained at least a 30%, 50% and 75% reduction in MMD, respectively (Fig. 3). No patient became completely migraine/headache-free during the treatment.

**Fig. 4:**
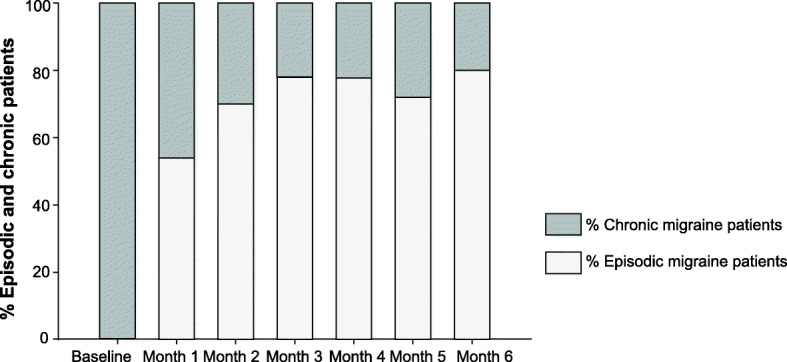
Percentage of chronic and episodic migraine patients: Percentage of patients who converted into an episodic migraine pattern (< 15 headache days/month) at each month, post-erenumab treatment initiation

A single dose of erenumab 70 mg led to a conversion from a CM pattern to an episodic pattern in 27% of our refractory CM patients. At month 3, the percentage of patients displaying an episodic migraine pattern was 39% and at month 6, it was 40% (Fig. 4).

At month 6, 60% of patients (*N* = 44/73) achieved at least 30% reduction in mean MMD and therefore continued the treatment. Conversely, 40% (*N* = 29 patients) did not reach the continuation threshold, hence the treatment was discontinued.

### Dose comparison

In 53 patients (53%) who did not achieve a clinically significant improvement with three monthly erenumab 70 mg injections, the dose was increased to 140 mg for the subsequent three months. Of these, 43/53 completed the 6 months treatment at the time of the analysis. Compared to baseline, 16/43 patients obtained > 30% but < 50% reduction in mean MMD and 27/43 patients obtained < 30% reduction in mean MMD at month 3. At month 6, erenumab at 140 mg significantly decreased mean MMD (− 3.6 vs – 9.8; *P* < 0.001) and mean MHD (− 4.1 vs – 9.2; *P* = 0.003), compared with data collected at month 3 after 3-monthly injections of Erenumab at 70 mg. Switching to 140 mg had no additional effect on headache free days (+ 2.2 vs + 5.5; *P* = 0.095), on number of abortive treatment intake days (− 3.8 vs – 4.9; *P* = 0.25) or on HIT-6 scores (− 2.8 vs – 3.9; *P* = 0.47). However, of the 27 patients who did not achieve a 30% reduction in mean MMD after three monthly injections with erenumab 70 mg, five patients (19%) achieved at least a 30% reduction in mean MMD at month 6, after increasing the dose to 140 mg for three consecutive months and hence continued the treatment further.

### Comparison of MOH and non-MOH patients

No significant differences in number of MMD, MHD and headache-free days emerged between the group of patients with MOH and that without MOH at baseline (Table 2). The percentage of patients with MOH was reduced from 54% at baseline to 20% after three treatment and to 25% after 6 treatments. Patients characterised as MOH at baseline had no significant differences in the reduction of migraine and headache days or HIT-6 score compared to non-medication overuse patients (nMOH) following three and six monthly injections of erenumab (Table 2).

**Table 2:** Clinical characteristics at baseline and after three and six treatments with erenumab for the patients who presented with medication overuse (MOH) and non-medication overuse (nMOH) at baseline, before treatment initiation

	Baseline		Month 3		Month 6	
	MOH	nMOH	MOH	nMOH	MOH	nMOH
**Migraine days**	19.0 ± 0.9	19.9 ± 1.2	12.4 ± 1.2*	14.7 ± 1.8	11.4 ± 1.3*	13.3 ± 2.2#
	*P* = 0.2		*P* = 0. 1		*P* = 0. 1	
**Headache Days**	23.2 ± 0.7	23.8 ± 0.8	15.3 ± 1.3*	19.5 ± 1.6#	15.7 ± 1.4*	18.2 ± 2.0
	*P* = 0.3		*P* = 0.1		*P* = 0. 1	
**Crystal clear headache days**	4.6 ± 0.6	2.7 ± 0.6	9.4 ± 1.2*	5.7 ± 1.4#	8.2 ± 1.4*	4.5 ± 1.5
	*P* = 0.1		*P* = 0.3		*P* = 0. 1	
**Days consumed abortives**	17.9 ± 0.7	4.0 ± 0.4	10.3 ± 1.0*	4.4 ± 0.6	9.7 ± 1.1*	3.8 ± 0.7
	*P* < 0.001		*P* = 0.1		*P* = 0. 001	
**HIT-6**	66.7 ± 1.0	66.7 ± 1.0	58.3 ± 1.3*	60.2 ± 2.2#	53.4 ± 3.1*	62.1 ± 4.3
	*P* = 0.8		*P* = 0.2		*P* = 0. 4	

*P < 0.05 compared to baseline values in MOH patients

# *P* < 0.05 compared to baseline values in nMOH patients

### Headache-related disability

Compared to baseline, the reduction of mean HIT-6 score was 7.7 points at month 3 (from 67.6 ± 0.4 to 59.9 ± 0.9) (*P* < 0.001) and of 7.5 points at month 6 (60.1 ± 1.3) (*P* = 0.01). The percentage of patients with severe headache-related disability was reduced from 96% at baseline to 68% after three monthly treatments and to 59% after six treatments. A percentage of 29% after three monthly treatments and 22% after six treatments reported some or little/ headache-related impact (Table 3).

**Table 3:** Changes in HIT-6 headache disability categories after three and six erenumab treatments

	Baseline (***N*** = 162) ***N*** (%)	Month 3 (***N*** = 100) ***N*** (%)	Month 6 (***N*** = 73) ***N*** (%)
**Severe impact (60–78)**	156 (96%)	68 (68%)	43 (59%)
**Substantial impact (56–59)**	5 (3%)	3 (3%)	14 (19%)
**Some impact (50–55)**	1 (1%)	15 (15%)	11 (16%)
**Little or no impact (< 48)**	0 (0%)	14 (14%)	5 (6%)

**HIT-6**, headache impact test-6; **N**, number

### Safety and tolerability

After one injection 48% (*N* = 77/162) of patients reported at least one side effect. Of them, the most frequent adverse events were constipation in 32 patients (42%) and flu/cold-like symptoms in 25 patients (32%). Twenty-two percent of patients (*N* = 22/100) at month 3 and 15% (*N* = 11) at month 6 reported at least one adverse. Table 4 outlines the changes of incidence of adverse events overtime in the whole population. Adverse events were transient, lasting up to two weeks post-injection and described as mild or moderate in the great majority of patients. However, 12% of patients (*N* = 19) discontinued erenumab due to severe adverse events, eight during months 1–3 and nine during months 4–6. The reasons for discontinuation were: severe constipation in nine patients, severe and consistent headache worsening after each injection in five patients, severe flu-like symptoms in two patients, whole body itchiness in one patient, severe mood deterioration in one patient and new onset hypertension in one patient. This patient developed raised BP while on monthly erenumab 70 mg and then 140 mg, confirmed with a 24-h BP monitoring and a cardiology assessment. A thorough cardiac work-up did not show any other underlying causes. Although a 75% migraine improvement was reported, the treatment was discontinued and the BP normalised within six weeks. No other patients demonstrated pathological changes to BP or heart rate during the first six months of treatment. One patient became pregnant during the first four months of treatment, hence the treatment was discontinued. Pregnancy was reported without complications.

**Table 4:** Percentage of incidences of adverse events at months 1, 3 and 6 following treatment with erenumab in the overall population of patients treated with erenumab

	Month 1 (***N*** = 162) ***N*** (%)	Month 3 (***N*** = 100) ***N*** (%)	Month 6 (***N*** = 73) ***N*** (%)
Constipation	32 (20%)	11 (11%)	4 (5%)
Cold-flu/like	25 (15%)	8 (8%)	2 (3%)
Generalised aches/pain	10 (6%)	1 (1%)	1 (1%)
Itchiness	8 (5%)	1 (1%)	1 (1%)
Injection site reaction (pain/skin redness)	5 (3%)	0 (0%)	1 (1%)
Muscle spasms	3 (2%)	0 (0%)	0 (0%)
Others	15 (9%)	4 (4%)	3 (4%)

**N**, number

## Discussion

This is the first large, independent, prospective analysis evaluating the effectiveness and tolerability of erenumab in real-world CM patients with and without MOH, refractory to medical treatments. Refractory CM is a very disabling migraine variant; it often represents a medical challenge for headache specialists and poses substantial burden on healthcare service utilisation [8]. The vast majority of patients treated in this audit would largely meet the recently EHF updated criteria for refractory CM since they failed all the drug classes with evidence in migraine prevention including injectable treatments and often non-invasive neuromodulation approaches, had severe migraine symptoms and reported high levels of headache-related disability [7]. Furthermore, a significant proportion of patients displayed a chronic daily headache pattern at baseline.

The results of this report suggested that over a period of six months, erenumab was well tolerated and effective in preventing migraine symptoms. Compared to baseline, erenumab led to a significant improvement across all the efficacy outcomes, which was sustained throughout the six months and led to a relevant reduction in headache-related disability. Our efficacy outcomes were less impressive than the ones of a recent real-life open-label study conducted predominantly CM patients [17]. Indeed, at month 6, 69% and 62% of patients obtained respectively at least 30% and 50% reduction in MMD. Similar outcomes were observed in the BoNT/A non-responder subgroup analysis. Possible explanation for the outcome differences between studies may include patients selection. In the Italian study, patients failed 2–4 treatments, hence were considered difficult-to-treat, whereas in our study most patients failed all established treatments, hence were more refractory to medical treatments. Furthermore, the increased proportion of responders at month 6 in the Italian study may have been influenced by the fact that non-responders could have discontinued the treatment earlier, whereas in our analysis, all patients, apart from those who discontinued because of adverse events, continued for the trial for six month, even if they did not respond at month 3.

The month-3 reduction in MMD with erenumab 70 mg reported in our analysis was similar to the main endpoint of the pivotal phase 2 CM clinical trial both when the whole study population was considered but also when the subgroup of patients who failed at least two preventive treatments was analysed [18, 19]. Furthermore, the 50% response rate with erenumab 70 mg in the overall Phase 2 trial population was 40% and in the subgroup analysis of patients with at least two prior treatment failures was 35.6%, very similar to the 35% response rate found in our patients. At month 6, a progressive improvement in most of the efficacy measures was observed in our patients, possibly due to the longer exposure to erenumab, but perhaps also due to the increased dose which may have enhanced the clinical improvement in some of our patients. A similar effect was reported in the 1-year open-label extension of the pivotal phase 2 clinical trial [20]. However, in that study, the withdrawal of treatment non-responders may have biased the results by impacting positively on the outcomes, whereas in our audit all patients were treated for at least six months unless they decided to discontinue it due to side effects.

Reduction of at least 30% in monthly migraine frequency is considered a clinically meaningful change especially in the refractory migraine population [21, 22]. If this cut-off was applied after three months treatment in our refractory patients, almost half of the patients (49%) would qualify for treatment continuation with erenumab. However, a small proportion of patients who did not obtain a 30% reduction in MMD at month 3, met the 30% threshold for treatment continuation at month 6, suggesting that highly refractory CM may benefit from a six month treatment, similarly to BoNT/A recommended regimen, to include those with a delayed response.

Along with the uncertainty about the optimal trial duration in refractory CM, it is also unclear whether the 140 mg erenumab dose is clinically superior to the 70 mg dose. In patients who switched from 70 mg to 140 mg, we observed a greater improvement in MMD and MHD. Furthermore a significant minority of non-responders after three monthly 70 mg erenumab injections, became responders once they were switched to the 140 mg dose, indicating a degree of superiority of the dose of 140 mg compared to the 70 mg. Similar findings emerged from the post hoc analysis of the pivotal erenumab CM study, which pointed towards a slight superiority of 140 mg dose in patients who failed two or more preventive treatments compared to those who were naïve or failed one treatment only [19], even when they had MOH [23]. Moreover, during the 1-year open-label treatment extension of the parent study, erenumab 140 mg showed greater clinical benefit compared to the 70 mg dose in a number of outcomes including reduction in MMD, 50%–75%–100% responder rates and reduction in days of use of abortive migraine medications [20]. Our data along with the post-hoc analysis of the pivotal CM trial, suggest that erenumab 140 mg dose may provide greater and more sustained efficacy compared to the 70 mg dose in the difficult-to-treat CM population.

Erenumab has led to a sustained resolution of MOH in a meaningful proportion of patients. Our data were similar to the outcome of the subgroup analysis of 274 CM patients with MOH treated with erenumab or placebo, showing no significant difference in treatment effect between the group with MOH and non-MOH [21]. Given that MOH is frequently diagnosed in tertiary referral clinics, the efficacy of erenumab in this even more complex group of patients, makes it a valuable option also in those patients in whom abortive treatment withdrawal is proven to be difficult to achieve or ineffective.

Erenumab has consistently shown a very favourable safety and tolerability profile across the CM and episodic migraine trials with low discontinuation rates [18, 24–26]. In the subgroup analysis of the pivotal phase 2 clinical trial, patients with prior preventive treatment failure treated with erenumab reported a higher proportion of AEs compared to placebo (42–58% depending upon the different doses) [19]. Our refractory group of patients displayed a similar proportion of AEs at month 1 (48%). However, with subsequent treatments, the proportion of patients complaining of AEs diminished substantially, suggesting that longer exposure to erenumab may lead to improved tolerability. Constipation and cold-like symptoms were confirmed to be the most frequent AEs, similarly to the pivotal clinical trials in migraine [18, 24–26]. However, the percentage of patients with constipation observed in our study was higher, especially during the first three months of treatment, compared to the one in the clinical trials. A likely explanation may be that we systematically asked about this adverse event every month for the first three months. However, a higher percentage of constipation (13.5%) was also observed in a recent real-life study [17], suggesting that the real-world population may be more susceptible to medications’ adverse events perhaps in view of their frequent co-morbidities. The discontinuation rate displayed by our patients was greater than the ones reported in previous studies, even when the subgroup analysis of the patients with prior preventive treatments failure was considered [18, 19]. Patients referred to our tertiary Centre have often been previously seen by multiple specialists across the country and tried many pharmacological options. High treatments discontinuation rates in the refractory CM patients may constitute one of the biological aspects of this complex migraine variant, especially in the context of multiple comorbidities, such as irritable bowel syndrome which was reported by a high proportion of our patients and may at least explain discontinuation due to severe constipation.

Hypertension has sparsely been associated to erenumab exposure [27]. In our patient no other causes of hypertension were identified. It is likely that their new-onset hypertension was erenumab-related given that no other causes were identified, the patient was otherwise healthy at baseline and the BP normalised with discontinuation of erenumab. In light of this case, it may be advisable to counsel patients about this possible and likely very rare AE.

The main limitation of this audit is the open label design. However, it is unlikely that the symptoms improvement could be explained by placebo alone. The strengths of this report are the large number of patients with a refractory form of migraine, which reflects the type of complex and difficult-to-treat patients seen in tertiary headache clinics and the prospective and the real-world nature of the analysis, which includes patients not subject to strict inclusion and exclusion criteria.

Recently published position statements and guidelines have debated patients selection and positioning of anti-CGRP MABs within the arsenal of migraine preventive treatments in clinical practice. Despite their meaningful efficacy and good tolerability demonstrated in clinical trials across the spectrum of episodic and chronic migraine, this new class of drugs is costly, hence in clinical practice it may be reserved for the difficult-to-treat/refractory subgroup of CM patients only, which were largely excluded for the clinical trials [28, 29]. Our analysis along with another recent study [17], provided the first real-world evidence of efficacy of erenumab in such population, supporting its meaningful role even in the complex difficult-to-treat CM population. In conclusion, erenumab was effective in the prevention of migraine symptoms in our refractory CM patients with and without MOH.

## Conclusion

Erenumab was effective in the prevention of migraine symptoms in our highly refractory CM patients with and without MOH. Erenumab’ s beneficial effect seems to be sustained, progressive overtime and not influenced by the level of patients’ refractoriness. The improvement in migraine symptoms led to a vast reduction in headache-related disability in our very complex group of patients. Treatment-related AEs reduced overtime, confirming its favourable tolerability profile. Hypertension emerged as a rare AE that needs to be taken into account when considering this treatment.

## Data Availability

Anonymized data are available from the Authors upon reasonable request.

## Abbreviations

AEs: Adverse events
BoNT/A: Botulinum toxin type A
BP: Blood pressure
CGRP: Calcitonin gene-related peptide
CM: Chronic migraine
EHF: European Headache Federation
EM: Episodic migraine
FDA: Food and Drug Administration
GONBs: Greater occipital nerve blocks
HIT-6: Headache Impact Test, 6th Edition
ICHD: International Classification of Headache Disorders
IHS: International Headache Society
MABs: Monoclonal antibodies
MHD: Monthly headache days
MMD: Monthly migraine days
MOH: Medication overuse headache
NHS: National Health System
NICE: National Institute for Health and Care Excellence
SD: Standard Deviation
SE: Standard error
U.K.: United Kingdom
